# Application of Optical Coherence Tomography in the Detection and Classification of Cognitive Decline

**DOI:** 10.5005/jp-journals-10028-1238

**Published:** 2018-03-01

**Authors:** Moon J Lee, Alison G Abraham, Bonnielin K Swenor, A Richey Sharrett, Pradeep Y Ramulu

**Affiliations:** 1 Medical Student, Wilmer Eye Institute, Johns Hopkins University School of Medicine, Baltimore, Maryland, USA; Department of Epidemiology, Johns Hopkins Bloomberg School of Public Health, Baltimore, Maryland, USA; 2 Associate Professor, Wilmer Eye Institute, Johns Hopkins University School of Medicine, Baltimore, Maryland, USA; Department of Epidemiology, Johns Hopkins Bloomberg School of Public Health, Baltimore, Maryland, USA; 3 Assistant Professor, Wilmer Eye Institute, Johns Hopkins University School of Medicine, Baltimore, Maryland, USA; Department of Epidemiology, Johns Hopkins Bloomberg School of Public Health, Baltimore, Maryland, USA; 4 Professor, Division of Cardiovascular Disease and Clinical Epidemiology Johns Hopkins Bloomberg School of Public Health, Baltimore Maryland, USA; 5 Associate Professor, Wilmer Eye Institute, Johns Hopkins University School of Medicine, Baltimore, Maryland, USA; Department of Epidemiology, Johns Hopkins Bloomberg School of Public Health, Baltimore, Maryland, USA

**Keywords:** Alzheimer’s disease, Cognitive impairment, Dementia, Literature review, Optical coherence tomography (OCT), Retinal nerve fiber layer.

## Abstract

**Aim:**

This review aims to critically analyze the current literature on the relationship of optical coherence tomography (OCT) measures to cognition and dementia.

**Background:**

Optical coherence tomography, a noninvasive method of imaging neuroretinal layers, and OCT angiography, a highly precise method of examining retinal vasculature, have widely been used to aid in the diagnosis and monitoring of a variety of ocular diseases. There is now an increasing body of evidence relating the structural and microvascular changes of the retina to cognitive impairment.

**Review results:**

In general, several studies have found decreased retinal nerve fiber layer (RNFL) thickness in Alzheimer’s disease (AD) and mild cognitive impairment (MCI) and an association between RNFL thickness and continuous measures of cognitive ability, though findings were inconsistent across studies. In many studies, associations were found for specific regions of the RNFL but not with overall thickness. Studies linking OCT measures to non-Alzheimer’s dementia were lacking, and limited work has been done on persons with past cognitive decline but who remain cognitively normal (the ideal stage at which to target treatment). Common limitations of prior studies include a failure to account for intraocular pressure (IOP) and axial length.

**Conclusion:**

Current research suggests a potential association between retinal findings observed on OCT and cognitive impairment. Methodologically robust research accounting for important covariates and looking at changes in OCT and/ or cognition is needed to better characterize the association between OCT and cognitive ability.

**Clinical significance:**

Further research is warranted to determine whether OCT findings can help identify the etiology of cognitive decline and/or serve as objective markers of AD. If this is the case, OCT may also help identify the presence of disease processes in cognitively normal individuals.

**How to cite this article: **Lee MJ, Abraham AG, Swenor BK, Sharrett AR, Ramulu PY. Application of Optical Coherence Tomography in the Detection and Classification of Cognitive Decline. J Curr Glaucoma Pract 2018;12(1):10-18.

## BACKGROUND

### Introduction

In 2015, the global burden of dementia was an estimated 46.8 million, a figure, that is expected to double by 2035.^1^ The disease process underlying dementia causes memory loss and cognitive decline in the aging population. The most common clinical dementia diagnosis is AD followed by vascular dementia (VaD). An increasing body of literature suggests that OCT may be used in patients with cognitive impairment to identify structural thinning of specific neuroretinal layers as a surrogate for neurodegeneration in the brain. While past studies have focused on structural OCT measures, recent advances allow OCT machines to also acquire a retinal angiogram through a technique known as OCT angiography, which detects submillimeter regions of nonperfusion that may be analogous to the microinfarcts seen in the autopsied brains of patients with either vascular or mixed dementia. The detection of structural and vascular changes using OCT may help address the challenge of establishing the etiology of dementia. Here, we review the literature associating structural retinal and retinovascular findings with cognitive impairment and discuss the clinical implications.

### Determining the Etiology and Future Course of Cognitive Impairment

Dementia may refer to any of a number of conditions including AD, VaD, as well as dementia with Lewy bodies (LBD), Parkinson’s disease dementia, or frontotemporal dementia (FTD). Distinguishing between these causes of dementia is often challenging due to mixed pathology, lack of noninvasive biomarkers of disease, heterogeneous phenotypes, and changing diagnostic criteria.^1,2^ The use of OCT offers the possibility that OCT-defined retinal parameters may implicate distinct etiology of cognitive impairment and enhance our understanding of the underlying mechanisms at play. Specifically, the retinal changes associated with distinct dementia profiles may help differentiate between the various etiologies of cognitive decline and aid in identifying individuals likely to develop more rapid cognitive decline allowing for earlier therapeutic intervention.^3^

Previous studies have shown high misdiagnosis rates of dementia type, with inconsistencies in clinical and neuropathological diagnosis, and VaD most often misdiagnosed as AD.^4,5^ Establishing a correct etiology is of importance for both prevention and treatment as the majority of therapeutic interventions currently in development are targeted toward a specific etiologic type of dementia.^6^ The gold standard for determining the etiology of cognitive impairment is neuropathology completed at autopsy.^6^ However, etiologic distinctions facilitating proper treatment must be made clinically. Additionally, a proper understanding of etiology helps predict the course of disease, including future mortality rates and rates of cognitive decline.^7,8^ If certain retinal findings are associated with specific etiologies, OCT may help improve the accuracy of etiologic dementia diagnosis, with clinical implications for cost as well as the management and treatment of disease. If this is the case, it may provide similar information as cerebrospinal biomarkers without the invasiveness of a spinal tap and more specific or augmentative information than cognitive tests.

It is particularly difficult to determine etiology early on in dementia processes, especially in persons who may be cognitively normal but have declined from their baseline. As a result, there have been significant efforts to identify biomarkers of AD which may identify persons likely to develop the disease, before significant cognitive deficits have occurred. In diagnoses of mild cognitive impairment (MCI), etiology is not a significant consideration and the diagnostic stability of MCI is even lower than in dementia, as the full-range clinical manifestations specific to the underlying cause of cognitive decline may not yet be apparent.^6^ This presents a clinically important challenge, as therapeutics aimed at slowing the neuro-degenerative process in specific forms of dementia likely need to be administered early in the disease course for maximal efficacy.^6^ If OCT can be shown to help identify the cause of cognitive decline and predict which patients are likely to develop future cognitive impairment, then it may have significant clinical value.

### Biomarkers of AD Pathologic Processes

One study that demonstrated the possibility of finding biomarkers for specific forms of cognitive impairment is the BIOCARD study. The study began in 1995 and aimed to identify novel biomarkers predicting early cognitive declines among individuals with normal cognition at baseline. They found that high baseline levels of cere-brospinal fluid (CSF) t-tau and/or p-tau combined with low baseline levels of CSF Ap1-42 were associated with decline in cognition among individuals who were cogni-tively normal at baseline.^9^ These CSF markers were then used to classify preclinical Alzheimer’s stages 0, 1, and 2^10^ *vs *those with suspected non-Alzheimer’s pathology. Preclinical disease stage 2 (defined by low CSF Api-42 and high CSF t-tau or p-tau) was associated with more rapid decline in a cognitive composite score than those in the other two categories.^9^ This study demonstrates the potential to use biomarkers to identify individuals at higher risk of cognitive decline, offering more opportunity for early therapeutic intervention. Despite this progress, however, there still exists a clear need to identify early disease makers that can be obtained through less invasive (and less costly) means.

### Retinal Changes and Cognitive Decline— Biological Plausibility

The biologic plausibility of the association between retinal pathology and brain pathology is based on shared embryology, anatomy, immunologic responses, disease pathology, and molecular findings across the retina and brain. During embryological development, both the retina and optic nerve extend from the central nervous system (CNS) developing from the diencephalon.^11^ Thus, the eye and brain microvasculatures share common physiology and susceptibility, while the blood-ocular barrier closely resembles the structure and properties of the blood-brain barrier.^11,12^ Shared properties across these two barriers include autoregulation, low-flow, and high-oxygen extraction.^12^ Hence, shared embryology suggests that the retina and CNS may be susceptible to the same disease processes. In addition, there are structural similarities between retinal ganglion cells (RGCs) and CNS neurons as well as the optic nerve fiber tract and other fiber tracts in the CNS. The RGCs also undergo the same neurodegenerative processes affecting directly injured neurons, resulting in degeneration of axons, glial scar formation, and loss of myelin.^11^ Following injury to the optic nerve, biochemical and metabolic changes create an environment that leads to secondary neurodegenerative processes in surrounding neurons.^11^ These responses to neuronal injury of the optic nerve are reflective of the secondary neurodegenerative changes that occur in the brain and spinal cord.^11^

Specific structural pathologic changes identified in CNS disorders have also been suggested to occur in retinal disease.^13^ Common pathogenic processes have been identified in AD, glaucoma, and age-related macular degeneration. These include reactive gliosis, inflammation, and oxidative and metabolic stress leading to neuronal death.^13^ Additional pathologic similarities in glaucoma and AD include axonal atrophy, deficits in axonal transport, and transsynaptic degeneration in the brain.^11^ At the molecular level, immunologic molecules and cytokines present in the CNS have also been identified in immune responses in the eye.^11^ Several of the aforementioned commonalities in the brain, spinal cord, and retina have been used to explain the retinal changes associated with AD, which include structural changes of the retinal microvasculature, structural changes in the optic nerve head (ONH), thinning of the RNFL, and, debatably, the presence of AD-associated proteins in the retina.^14^

Findings from autopsy specimens support the presence of different mechanisms underlying the various dementia entities and raise the possibility that OCT measures examining retinal features may be capable of distinguishing persons with these varying cerebral disease mechanisms *in vivo. *Neuropathological changes reported in AD include amyloid plaques, neurofibril-lary tangles, glial responses, and neuronal and synaptic loss.^15^ In addition to these molecular findings, AD has also been associated with changes in brain volume of the frontal subcortical region with subsequent involvement of temporoparietal cortical structures.^16^ Such changes in brain volume may also be reflected by eye neural volumes (i.e., RNFL thickness) in neurological structures, as demonstrated in other neurologic disease processes.^17^ Vascular disease also plays a substantial role in cognitive decline; both VaD and common mixed pathologies^18^ result to varying degrees from vascular disease, and similar vascular pathology has been observed in the retina. The vascular defects most strongly associated with cognitive impairment are submillimeter infarcts which are not radiographically visible and, up until now, have only been detectable at autopsy.^19^ Optical coherence tomography angiography, which can detect such microvascular changes in the retina, offers a potential way to measure the vascular disease processes occurring in a closely related vascular bed. Thus, OCT, as a single modality, offers the possibility of measuring several factors (i.e., thickness of various retinal layers, vascular density within various beds) that may help identify the reasons for dementia. If so, OCT findings might serve as biomarkers of the specific disease processes underlying cognitive impairment.

### Retinal Photography: Early Evidence of Retinal Changes in Cognitive Decline

Even before sophisticated retinal measures could be acquired using OCT, early studies utilizing retinal photography suggested that ganglion cell (GC)/optic nerve damage could mark cognitive impairment.^20,21^ Tsai et al^20^ evaluated differences in the RNFL, disk pallor, cup-to-disk ratio, cup volume, and rim area in AD *vs *healthy controls and found a potential for using these parameters to monitor the progression of AD. They found that higher pallor, increased cup-to-disk ratio and cup volume, and decreased disk rim area all correlated significantly with higher Alzheimer’s Disease Assessment Scale (ADAS) scores, raising the possibility that AD processes were occurring concurrently in the eye.^20^

The Atherosclerosis Risk in Communities (ARIC) study investigated the association between retinal vascular changes and cognitive decline. Study visits occurred every 3 years between 1987 and 1998, and tests included retinal photographs and cognitive function assessment. In 2002, the ARIC study reported that retinopathy was independently associated with lower cognitive function in middle-aged individuals without a history of stroke.^22^ The ARIC study also found that retinopathy was associated with cerebral atrophy noted by ventricular enlargement on magnetic resonance imaging.^23^ Following the ARIC baseline study, a subset of participants were then followed over a period of 14 years. This study found that retinal microvascular changes on retinal photographs were associated with cognitive decline, specifically declines in executive function and psychomotor speed, a finding that persisted even when diabetics were excluded from the analysis.^21^ These early longitudinal studies suggest the potential for use of OCT angiography to evaluate retinal microvascular changes as a possible predictor of cognitive decline.

### Precedent for use of OCT in Monitoring Disease

Optical coherence tomography has been used to image and measure the retina, RNFL, macula, optic nerve, and the anterior chamber. It is a noninvasive, high-resolution imaging technique that provides cross-sectional images of the retina and is increasingly being used to aid in the diagnoses and monitoring of a variety of diseases not limited to the eye, including neurologic disease.^24,25^ The use of OCT in multiple sclerosis (MS) offers a strong precedent for using OCT parameters to define severity and progression of neurological diseases. The NFL and ganglion cell complex (GCC) thinning are strongly associated with MS, and with brain substructure volumes relevant to MS.^26^ Of note, these results were found in patients without optic neuritis, i.e., in persons who were asymptomatic with regard to their vision.^26^ In longitudinal studies, GCC thinning was noted to be significantly greater among patients with active MS than stable MS.^27^ A recently published longitudinal study also found an association between rates of atrophy of the GC and inner plexiform layer and global neurodegeneration, and more specifically rates of atrophy of the whole brain, white matter, gray matter, and thalamus.^28^ As a result, OCT is increasingly being used to gauge MS progression and has been used as an objective secondary measure in a clinical trial for relapsing/remitting MS.^29^

## REVIEW RESULTS

### Use of OCT in Detecting Damage to the RNFL and Other Retinal Structures in AD

Most studies investigating OCT measures and cognition have focused on group comparisons of RNFL thickness in persons with significant cognitive loss (or diagnosis of AD) and persons with normal cognition. Several OCT studies have found decreased RNFL thickness in AD *vs *controls.^30-33^ Of these, a number of studies have found overall thinning (integrating data from all quadrants),^31,32^ while others have found a greater reduction in thickness of the inferior quadrant,^34^ or selective thinning of the superior quadrant.^35^ In addition to changes in RNFL thickness in AD, several studies have also reported on the presence of other retinal changes correlated with cognitive decline. Studies have detected a significant decrease in the combined RNFL and GC layer within the macular region^31^ and reduced mean total macular volume^32^ compared with healthy controls.

Studies examining OCT parameters in association with continuous measures of cognitive function have found conflicting results. Iseri et al found that total macular volume and Mini Mental Status Exam (MMSE) scores were significantly correlated in AD.^32^ The EPIC-Norfolk study also reported an association between RNFL thickness (measured by Heidelberg Retina Tomograph) and cognitive test scores assessing global function, recognition, learning, episodic memory, and premorbid intelligence in a population of older British adults.^36^ In contrast, others have found that OCT measurements were not correlated with MMSE, ADAS-Cognitive subscale, and Clinical Dementia Rating (CDR) evaluations.^37^ This lack of consensus in the association between OCT measurements and cognitive function may in part be due to the inconsistency in the types of cognitive function tests administered. Based on whether cognitive measurements assess global cognitive function or specific subdomains of cognition, associations with OCT measurements may vary.

In addition, there is also a lack of consensus on which retinal measurements (i.e., peripapillary RNFL, macular thickness, macular volume, etc.) are most likely to reflect AD. One study by Larrosa et al^38^ combined several of these OCT measurements to calculate and validate a linear discriminant function (LDF) for OCT and demonstrated decreased RNFL thickness in AD. Their LDF, which combined OCT measurements (circumpap-illary RNFL thickness and retinal measurements), had better diagnostic ability compared with individual OCT parameters. The best parameter for distinguishing AD patients from healthy subjects was the Spectralis RNFL LDF.^38^ This demonstrated the capacity for using OCT as a potential diagnostic tool in AD and raises the question of which retinal measurements should be made in association with cognitive function.

### OCT Findings in Early Cognitive Impairment prior to AD

If OCT is to be clinically useful, it will need to contribute to the identification of individuals likely to develop cognitive decline as a result of AD pathologic processes which are operative prior to dementia, i.e., persons with MCI due to AD, in whom treatment is most important. Indeed, several previous studies have investigated OCT measures in group comparisons of persons with MCI and healthy controls with normal cognitive function. Several studies have found that the overall RNFL thickness was significantly decreased in AD and MCI compared with healthy controls.^39-44^ However, data on regional selective thinning of the RNFL in AD and MCI are varied. One study found a significant reduction in RNFL thickness in AD and MCI compared with controls in all four quadrants,^40^ while another study found a significant decrease in RNFL thickness in only the superior and inferior quadrants in AD and MCI.^45^ Kesler et al^43^ found a significant decrease in RNFL in the inferior quadrants of patients with AD and MCI compared with controls. In addition, Shen et al^46^ found that in patients with MCI, inferior quadrant RNFL thickness was inversely associated with better cognitive function, reflected in higher episodic memory scores.

Other studies have compared additional retinal measures in AD patients, patients with MCI and controls; these measures include macular volume, choroidal thickness, and ganglion cell-inner plexiform layer (GC-IPL). One investigation showed decreased macular volume in AD and MCI compared with controls,^41^ though another found that MCI patients had a greater macular volume, followed by healthy controls, then AD patients.^44^ Bulut et al^47^ found significantly reduced choroidal thickness in AD and MCI in all investigated regions including sub-foveal, temporal, and nasal regions, and Cheung et al^42^ found significantly reduced GC-IPL in AD and MCI. Thus, studies investigating changes in OCT measures have found RNFL thinning in MCI similar to that reported in AD, but regional selectivity and other retinal changes in MCI remain varied.

Studies investigating the association between OCT measures and continuous measures of cognition in the disparate groups of AD *vs *MCI *vs *controls have reported conflicting results. Some have found no correlation between MMSE scores and thickness of RNFL or macular volume.^41^ In contrast, others have found significant correlations between MMSE scores and RNFL thickness,^39^ as well as choroidal thickness at all locations.^47^ One study comparing RNFL thickness in AD *vs *LBD *vs *Parkinson’s disease dementia found that MMSE scores and CDR scores correlated with thickness of RNFL in all three types of dementia.^48^ No studies were identified which investigated persons with disease even prior to MCI (i.e., persons with documented cognitive decline) but who still did not meet the criteria for MCI or dementia.

### OCT Measures as a Predictor of Cognitive Decline

A limited number of studies have investigated OCT as a predictor of future cognitive decline. Shi et al^34^ followed 78 participants categorized into two groups based on stable cognitive function *vs *presence of cognitive deterioration over a period of 25 months. Participants whose cognitive status deteriorated had a greater reduction (negative change) in RNFL thickness in the inferior quadrant, compared with stable subjects.^34^ Conversely, they found greater attenuation of RNFL thickness in the superior quadrant in subjects who maintained stable cognitive function.^49^ The results of these studies suggest the potential for OCT measurements to predict future cognitive impairment, the goal of current AD biomarker development efforts.

## DISCUSSION

### Methodological Challenges

The scope of this review is limited as there are few or no studies investigating structural or angiographic OCT measures in other etiologies of dementia, such as LBD, VaD, FTD, and Parkinson’s disease dementia. In the absence of these particular studies, it is unclear whether differential OCT retinal findings exist in association with specific etiologic forms of dementia and whether the OCT findings noted in the context of AD are universally found in all persons with dementia. In addition, the presence of vascular changes which may present in both AD and VaD adds complexity in our ability to potentially distinguish clear etiology using OCT findings. No studies have utilized OCT angiography to evaluate retinal vascular damage in any form of cognitive impairment, though this topic is a promising area for future research.

Prior studies on the investigation of AD and RNFL and GCC thinning are clouded by several methodological issues. First, all studies use clinical diagnosis of AD with no neuropathologic confirmation, making it more difficult to establish a valid association of neural OCT findings with AD. In addition, it is unclear whether AD damage to GCs and the RNFL is diffuse or focal. If damage is indeed focal, it remains unclear which regions may be more severely affected, and how this regionality is best established as fact as opposed to an artifact of multiple comparisons. Several of the aforementioned studies found that the RNFL in certain quadrants may be differentially affected in AD^34,35,43,45^; however, the rationale behind the preferential effects of a neurodegenerative brain disease on a specific region of the retina has yet to be established, raising the question of whether specific analyses of these regions reflect any prior hypothesis, or simply a chance finding generated by employment of multiple comparisons.

The current method of comparison of NFL thickness maps to that of age-matched healthy controls in specific sectors presents with methodological challenges. Expected peaks do not necessarily coincide with peaks in thickness for every individual’s NFL map. Possible explanations for this phenomenon include the temporal peak shift in myopic individuals and the nasal peak shift in hyperopic individuals. In addition, if focal damage to the RNFL is indeed occurring in the context of Alzheimer’s, one potentially useful methodological approach may be to analyze the point of greatest difference between the expected and observed values in the ONH, customizing expected thickness at each point around the ONH with all available information. Thus, by using customized NFL thickness maps or other innovative approaches, investigators could focus on measuring changes in the most heavily affected area of the retina to investigate the potential focal effects of AD.

In addition to the possible focal effects of AD on the RNFL, it is also possible that only specific retinal cell types are affected in AD and other brain diseases. For example, research shows changes in specific retinal layers in different subtypes of spinocerebellar atrophy.^50,51^ In particular, the GCC-IP complex in the macula has been associated with AD.^42^ Based on the potential differential effects of AD on specific types of retinal cells, there are different OCT measures that could be used to identify and track AD. If AD-related damage is most strongly associated with GC damage within the macula, then macular GC-IPL thickness would serve as an appropriate measure of the retinal changes in AD.^52^ Conversely, if GCs throughout the retina are damaged in persons with AD, this may be best evaluated in measurements of the peripapillary retinal nerve layer, which reflects all axonal projections leaving the retina to join the optic nerve. Thus, further investigation into the association of AD and RNFL thinning is needed to determine the potential focal and cell-specific effects of AD.

**Fig. 1:**
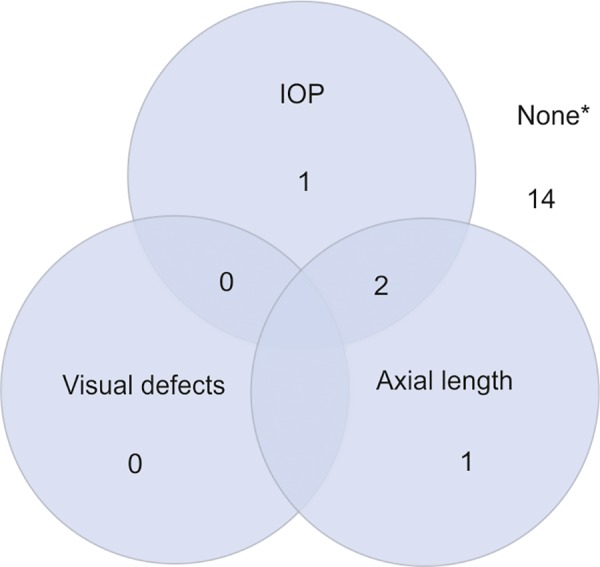
Variables accounted for in previous studies; *Variables were considered accounted for if directly measured and statistically compared between groups. Of the studies that did not include these variables in their statistical analysis, 12 included one or more of the listed variables as exclusion criteria^30-32,34,35,38,39,41-43,45,48^

In order to more effectively clarify this relationship between OCT measures and cognition, studies should also take into account variables that could potentially affect the association between cognition and OCT measures (particularly RNFL thickness). Intraocular pressure may affect the precision of association measures as it is a strong risk factor for glaucoma and its associated GC loss.^53-59^ Thus far, these variables have been inconsistently measured in clinical studies (Fig. 1). For example, IOP is only assessed in one study relating GCC or RNFL thickness to cognitive outcomes.^38^ In studies that have not accounted for IOP, differences in GC numbers may be due to the differential effects of IOP rather than a shared eye/brain process of neural damage. In addition, few studies have adjusted for axial length. In the presence of greater axial length, RNFL thickness measures may be incorrect due to magnification effects leading to an artifactual minification of the image.^60-63^ This would result in a smaller observed measurement of the RNFL. Increased axial length is also associated with more education and time spent reading during childhood,^64^ and this difference in educational background may strongly affect cognitive scores.^65^ Failure to adjust for axial length as a confounding variable may skew the association of RNFL thickness and cognitive outcomes, suggesting an association between decreased RNFL thickness and better cognitive function.^57,58^ Cognitive testing results may also be affected by visual defects (i.e., difficulty distinguishing the lines in the Trails test), which would be expected to accompany thinning of the RNFL, as opposed to true cognitive impairment. Thus, visual function may confound the association between OCT measurements and cognitive testing outcomes. Studies may account for this by conducting longitudinal studies and mediation analyses to estimate direct and indirect (mediated by vision) effects of OCT measures. This will yield estimates of decline in cognitive testing scores attributed to vision loss separate from estimates in decline due to degeneration of the RNFL. Studies may also utilize nonvisual cognitive assessments, so estimates of cognitive function are less impacted by visual deficits.

**Table 1:** **Table 1: **Priorities for future work

• Assessment of OCT findings in non-AD forms of dementia	
• Integration of angiographic measures to explore vascular contribution	
• Use of customized NFL thickness maps to investigate the potential focal effects of AD	
• Consistent incorporation of IOP, axial length, and other visual defects when evaluating statistical considerations	
• While understanding the impact of OCT with cognitive measures, consideration for the fact that cognitive measures are largely assessed through visually demanding tests (potential for the use of non-visual cognitive assessments)	
• Longtudinal studies on OCT measures and cognitive change in dementia	

Other limitations in the current literature include failure to have OCT images read at an established reading center leading to the inclusion of artifacts (i.e., retinal layer missegmentation) which may have contributed to producing erroneous values^41^ and the variation in measurement differences in assessing cognitive and retinal changes. Due to the limited accuracy of the clinical diagnosis of dementia, OCT measures must be investigated in the setting of a proven etiology of cognitive impairment in order to assess its potential use as a diagnostic tool. Future studies may include the validation of RNFL thickness attenuation against known biomarkers of dementia or positron-emission tomography scans and pathology. Finally, the majority of previous studies were cross-sectional, making it difficult to establish temporality (Table 1). The observed retinal findings may have preceded cognitive changes, occurred simultaneously, or even occurred after pathologic changes in the brain due to dementia. This temporality is important to determine whether OCT can be used to aid in the early detection of dementia.

## CONCLUSION

Despite the methodological challenges in assessing the use of OCT in dementia, this technology offers a potentially objective, noninvasive method of determining the etiology of cognitive decline, identifying persons with early-stage disease, and predicting which persons are at greater risk for future cognitive decline. Current research showing structural and physiological similarities between the retina and CNS, as well as more specific retinal findings in AD, suggests a potential association between retinal changes and cognitive impairment due to AD or other cerebral neurodegenerative conditions. Thus, OCT measures may serve as a marker of the different pathogenic processes underlying cognitive impairment and dementia type. Further work is needed to determine whether structural OCT measures can specifically distinguish AD-related cognitive impairments to non-AD changes and whether OCT can predict individuals who are likely to go on to develop more rapid cognitive loss. Optical coherence tomography angiography should also be further investigated to determine whether it can be useful in identifying persons with specific types of dementia where vascular disease is relevant (i.e., VaD and possibly AD). Future methodologically robust research is needed to clarify the possible focality of retinal damage caused by cognitive impairment, and longitudinal studies accounting for potential confounders are needed to determine the temporality of the possible association between retinal changes and dementia.

## CLINICAL SIGNIFICANCE

### Implications

The use of OCT measurements as objective biomarkers of dementia type and rapid progression due to AD has several implications for both the fields of ophthalmology and neurology. First, it adds complexity to the monitoring of ocular diseases. There are several ocular conditions, such as glaucoma, in which a reduction in RNFL thickness is attributed to a glaucomatous process. There is also an expected reduction in the number of GCs with aging.^66^ However, the studies discussed above illustrate that other systemic factors add variability to the accepted normative level of GCL loss. Hence, in individuals with both ocular disease and systemic comorbidities, there may be an expected level of age-related GCL loss superimposed upon GCL loss caused by systemic factors and ocular diseases. The same complexity would apply for other neuro-ophthalmologic disease processes. Consideration must be given to the idea that other systemic processes may affect metrics which we have traditionally considered solely to reflect local disease processes. These implications make it increasingly difficult to quantify and accurately attribute changes in RNFL thickness to a glaucomatous process, neuro-ophthalmic condition, or a systemic neurocognitive disease.

The use of OCT to measure biomarkers in pathologic processes underlying dementia and cognitive decline also has implications in the practice of neurology. Optical coherence tomography measures may even be used as an objective noninvasive biomarker to follow progression of other neurologic diseases, such as MS.^27,67,68^ Future work will determine whether OCT measures may be useful in the field of cognition if the demonstrated retinal changes prove to be true in the context of cognitive impairment.
